# Protein Tyrosine Phosphatase µ (PTP µ or PTPRM), a Negative Regulator of Proliferation and Invasion of Breast Cancer Cells, Is Associated with Disease Prognosis

**DOI:** 10.1371/journal.pone.0050183

**Published:** 2012-11-20

**Authors:** Ping-Hui Sun, Lin Ye, Malcolm D. Mason, Wen G. Jiang

**Affiliations:** Metastasis & Angiogenesis Research Group, Institute of Cancer and Genetics, Cardiff University School of Medicine, Cardiff, United Kingdom; Karmanos Cancer Institute, United States of America

## Abstract

**Background:**

PTPRM has been shown to exhibit homophilic binding and confer cell-cell adhesion in cells including epithelial and cancer cells. The present study investigated the expression of PTPRM in breast cancer and the biological impact of PTPRM on breast cancer cells.

**Design:**

Expression of PTPRM protein and gene transcript was examined in a cohort of breast cancer patients. Knockdown of PTPRM in breast cancer cells was performed using a specific anti-PTPRM transgene. The impact of PTPRM knockdown on breast cancer was evaluated using *in vitro* cell models.

**Results:**

A significant decrease of PTPRM transcripts was seen in poorly differentiated and moderately differentiated tumours compared with well differentiated tumours. Patients with lower expression of PTPRM had shorter survival compared with those which had a higher level of PTPRM expression. Knockdown of PTPRM increased proliferation, adhesion, invasion and migration of breast cancer cells. Furthermore, knockdown of PTPRM in MDA-MB-231 cells resulted in increased cell migration and invasion via regulation of the tyrosine phosphorylation of ERK and JNK.

**Conclusions:**

Decreased expression of PTPRM in breast cancer is correlated with poor prognosis and inversely correlated with disease free survival. PTPRM coordinated cell migration and invasion through the regulation of tyrosine phosphorylation of ERK and JNK.

## Introduction

Protein tyrosine phosphatases (PTPs) consist of a large family of related enzymes. Classical PTPs include transmembrane receptor-like protein (PTPRs) and non-transmembrane PTPs. PTPs play a profound role in many cellular functions including cell survival, proliferation, differentiation, adhesion and motility. The classical PTPs participate in regulation of apoptosis via several pathways such as the nuclear factor kappa B (NFκB) pathway, extracellular signal regulated kinase (ERK) pathway, PI3K/Akt pathway (SHP2) and p53 pathway (TC-PTP) [1]. PTPs are characterised by variable extracellular multiple domains and exhibit features of cell-adhesion molecules in their extracellular segment. And it have been implicated in cell-cell and cell-matrix contact via dimerisation, phosphorylation and reversible oxidation [2], [3]. Furthermore, certain forms of PTPs have been shown to be a potential target for bisphosphate in the treatment of bone metastasis from cancer. Deregulation of these pathways has been implicated in cancer development and progression [4].

To date, evidence has demonstrated that PTPs function as tumour suppressors and they also play an important role in other diseases. For example, PTEN (MMAC1) is a tumour suppressor and its mutation has been found in many different human cancers and Cowden disease. PTEN coordinates cell proliferation and survival by suppressing the PI3K pathway. Deregulation of the PI3K-PTEN pathway can result in tumourigenesis [5], [6]. Moreover, mutation of PTPRJ (DEP1) has been indicated in breast, lung, thyroid and colon cancers [7], [8], [9], [10]. Furthermore, mutation of SHP2 (PTPN11) has been implicated in leukaemia and other human cancers with increased activity of oncogenic protein tyrosine kinases [8], [11], [12]. Additionally, a tumour suppressor function has been indicated for PTPκ (PTPRK) in primary central nervous system lymphomas [13], and PTPδ (PTPRD) in laryngeal squamous cell carcinoma and other human cancers [14].

Protein tyrosine phosphatase µ (PTP µ or PTPRM) has a similar structure to cell-cell adhesion molecules and has been shown to exhibit homophilic binding and confer cell-cell adhesion in cells including epithelial and cancer cells. Moreover, PTPRM also recognises other subfamily members to mediate cell-cell aggregation [15], [16]. Like other PTPs, PTPRM is regulated by the balance between the actions of protein tyrosine kinases (PTKs) and PTPs. PTPRM associates with E-cadherin/α-catenin/β-catenin complexes in rat heart, lung and brain tissues. In fact, PTPRM directly binds to the intracellular domain of E-cadherin rather than α-catenin and β-catenin to regulate the phosphorylation of the E-cadherin adhesion molecule and subsequently control its function [17], [18]. Furthermore, reduced expression of PTPRM resulted in an increased phosphorylation of tyrosine 992 of EGFR (pY992) by EGF, a docking site for phospholipase C γ 1 (PLCγ1) to activate PLCγ1, thus leading to increased cell migration in both wounding and chemotaxis assays [19], [20].

**Table 1:** Primer sequences used in current study.

Molecular	Forward primers (5′–3′)	Reverse primers (5′–3′)
PTPRM	TGCAGGAAACCATCTATGA	CTCTGTTTGCACCATGTTC
PTPRM (Q-PCR)	CCTTCTCCCCTATAAAAGCTA	ACTGAACCTGACCGTACAGCCACTTGGACACAGTCTAT
GAPDH	GGCTGCTTTTAACTCTGGTA	GACTGTGGTCATGAGTCCTT
GAPDH (Q-PCR)	CTGAGTACGTCGTGGAGTC	ACTGAACCTGACCGTACAGAGATGACCCTTTTG
MMP9	AACTACGACCGGGACAAG	ATTCACGTCGTCCTTATGC

Currently, the role played by PTPRM in cancer remains unknown. The present study aimed to examine the expression of PTPRM in breast cancer and its association with the disease progression and also the impact of this molecule on breast cancer cell functions.

## Materials and Methods

### Cell Lines and Cells Culture

Human breast cancer cell lines, MDA-MB-231, MCF-7, and ZR751 were obtained from the European Collection of Animal Cell Cultures (ECACC, Salisbury, England). Cells were routinely cultured with Dulbecco’s modified Eagle’s medium containing 10% foetal calf serum and antibiotics at 37°C with 5% CO_2_.

**Table 2:** Primary antibodies used in current study.

Protein target	Cat. number
ERK	sc-93
JNK	sc-571
PLCγ	sc-31748
p-Tyr	sc-508

**Figure 1:**
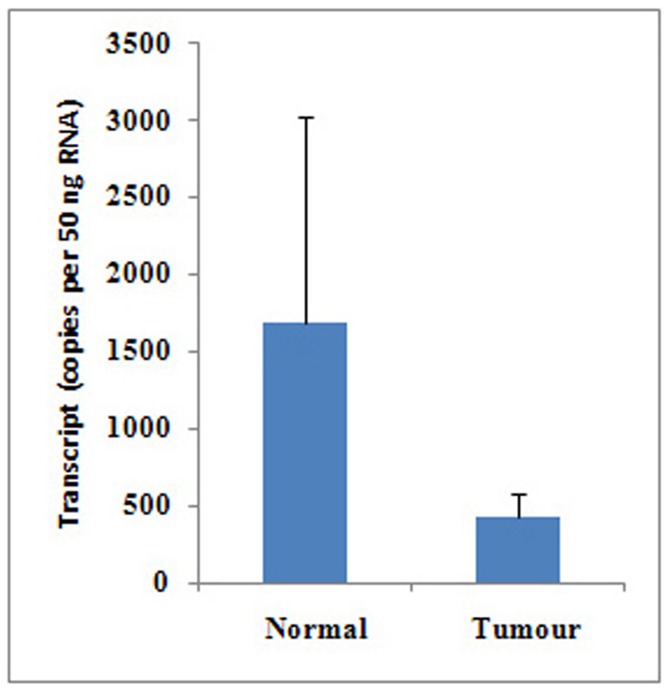
Expression of PTPRM in breast cancer tissues. The PTPRM transcript level was decreased in human breast cancer compared with normal breast tissues using quantitative PCR, *p* = 0.36.

### Human Breast Specimens

A total of 160 breast samples were collected immediately after surgery and stored at −80°C until use, with approval of the Bro Taf Health Authority local research ethics committee. All patients were informed and participated with written consent. The cohort included 127 breast cancer tissues and 33 background normal breast tissues. All the specimens were verified by a consultant pathologist. A routine follow-up was carried out after surgery. The median follow-up period was 120 months. The clinical data is provided in **Table S1**.

**Figure 2:**
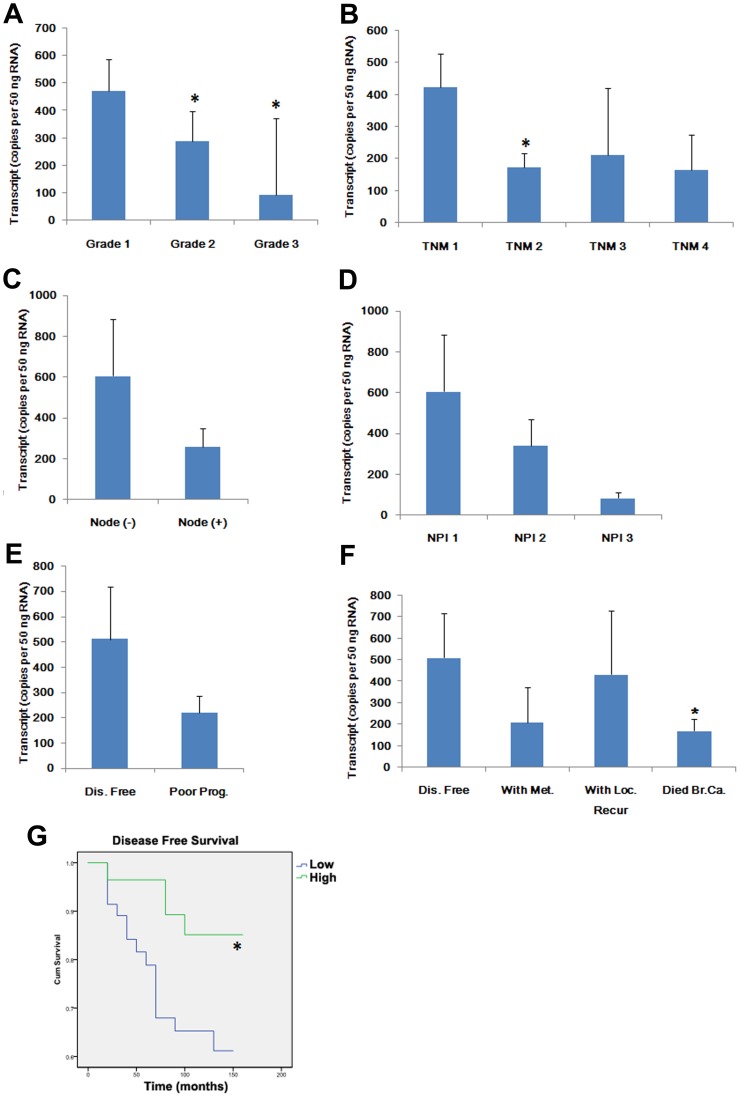
PTPRM, link to tumour grade, nodal status, TNM staging and clinical outcomes of breast cancer. A, PTPRM transcripts were decreased in the moderately and poorly differentiated cancer cells in comparison with well-differentiated tumour cells. B, lower levels of PTPRM transcripts were seen in the advanced breast cancer. PTPRM levels were higher in tumours of early TNM stage and were decreased in the TNM2, TNM3 and TNM4. C. decreased PTPRM expression was associated with lymphatic metastasis but this was not statistically significant. D, PTPRM and Nottingham Predictive Index (NPI). NPI 1 group (NPI score<3.5; n = 59) and NPI 2 group (NPI score = 3.5–5.4; n = 35), and NPI 3 group (NPI score>5.4; n = 15) represented patients with good, moderate, and poor prognosis, respectively. E, PTPRM expression was decreased in patients with poor prognosis including local recurrence, metastasis and death from the disease. F, PTPRM expression was significantly decreased in patients who died from the disease compared with that of disease-free patients. G, Reduced PTPRM transcript levels were correlated with poorer disease free survival. The average transcript level of PTPRM in NPI 2 group was used as a threshold. *, *p*<0.05.

**Figure 3:**
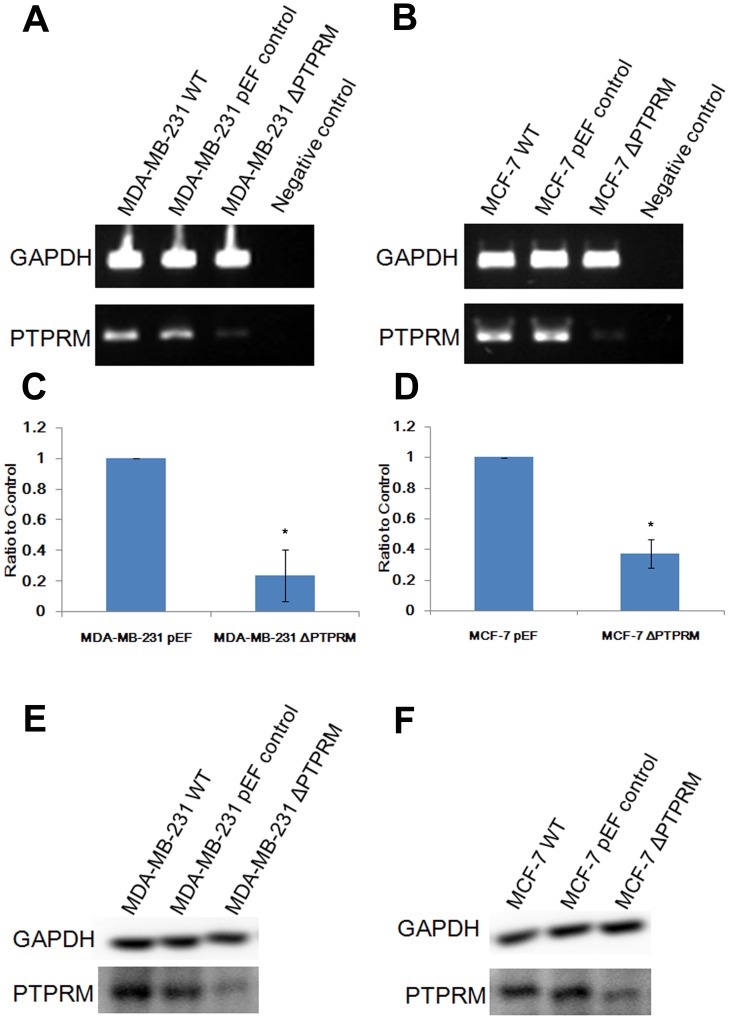
Knockdown of PTPRM in breast cancer cells. A and B, knockdown of PTPRM was seen in both MDA-MB-231^ΔPTPRM^ (A) and MCF-7^ΔPTPRM^ (B) cells using RT-PCR compared with their wild-type (MDA-MB-231^WT^ and MCF-7^WT^) and empty plasmid control (MDA-MB-231^pEF^ and MCF-7^pEF^) cells. C and D, knockdown of PTPRM in MDA-MB-231^ΔPTPRM^ (C) and MCF-7^ΔPTPRM^ cells (D) was also verified using real-time quantitative PCR compared with pEF control cells. E and F, knockdown of PTPRM in MDA-MB-231^ΔPTPRM^ (E) and MCF-7^ΔPTPRM^ cells (F) was confirmed using western blot in comparison with pEF control. *, *p*<0.05.

### Reverse Transcription-PCR

Total RNA extraction from frozen tissues and culture cells was performed using Tri Reagent (Sigma-Aldrich Inc, USA). Following reverse transcription into cDNA, PCR was carried out using ReadyMix PCR Reaction Mix (Sigma-Aldrich Inc, USA). Primer sequences are shown in **Table 1**. Reactions were carried out at the following conditions: 94°C for 5 min, 30 cycles of 94°C for 30 sec, 55°C for 30 sec and 72°C for 30 sec, followed by a final extension of 7 min at 72°C. PCR products were separated on a 1.5% agarose gel and photographed after staining with ethidium brominde.

**Figure 4:**
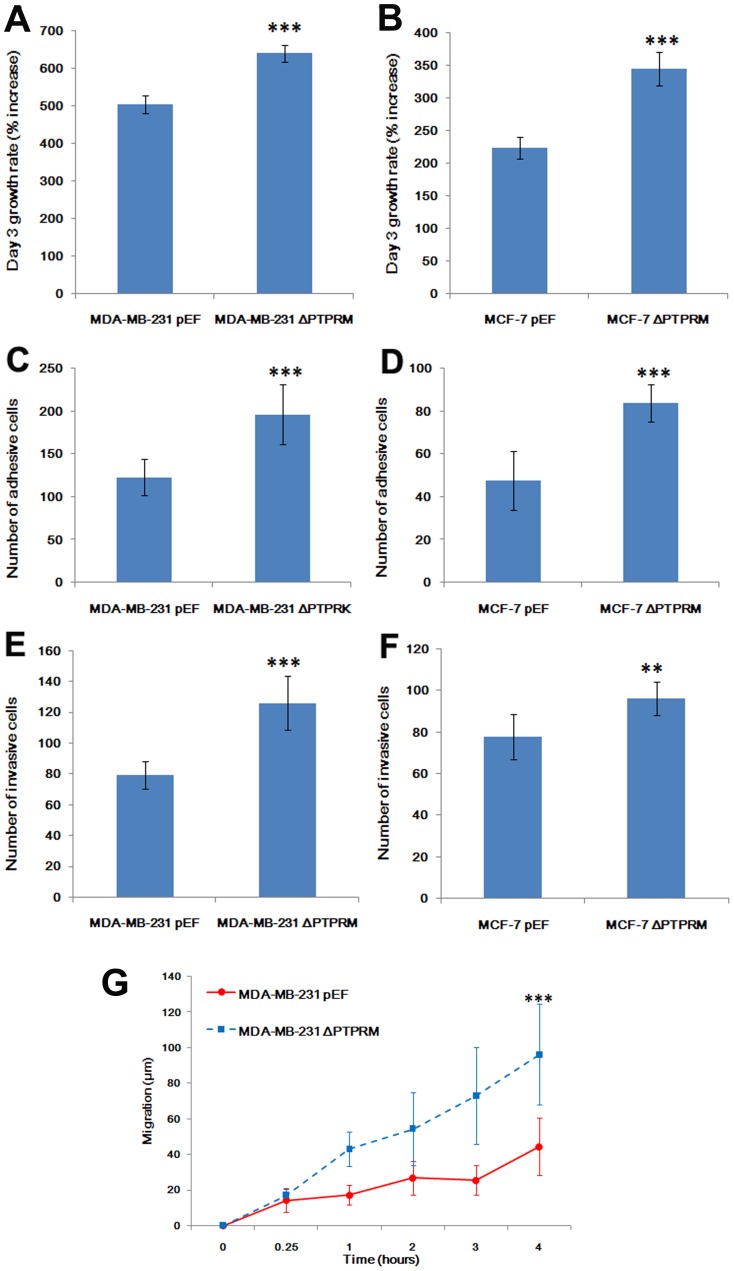
The effects of PTPRM knockdown on biological functions of breast cancer cells. A and B, Knockdown of PTPRM increased the *in vitro* growth of breast cancer cells. C and D, Knockdown of PTPRM promoted cell-matrix adhesion in both MDA-MB-231 and MCF-7 cells. E and F, Invasiveness of both MDA-MB-231 and MCF-7 cells were also promoted after knockdown of PTPRM. **, *p*<0.01 and ***, *p*<0.001.

**Figure 5:**
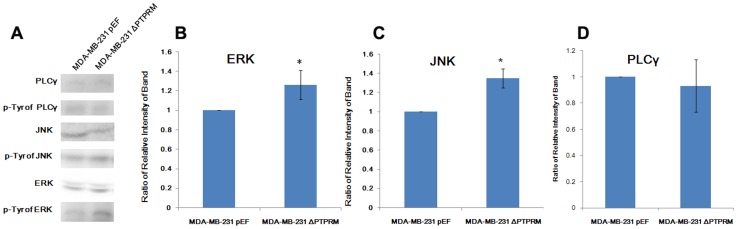
Impact on tyrosine phosphorylation of JNK and ERK. A, immunoprecipitation and western blot showed tyrosine phosphorylation of JNK and ERK were increased in PTPRM knockdown cells, which exhibited no effect on PLCγ phosphorylation. Relative intensity of bands from three western blots was analysed using Image J software for PLCγ (B), JNK (C), and ERK (D). *, *p*<0.05.

**Figure 6:**
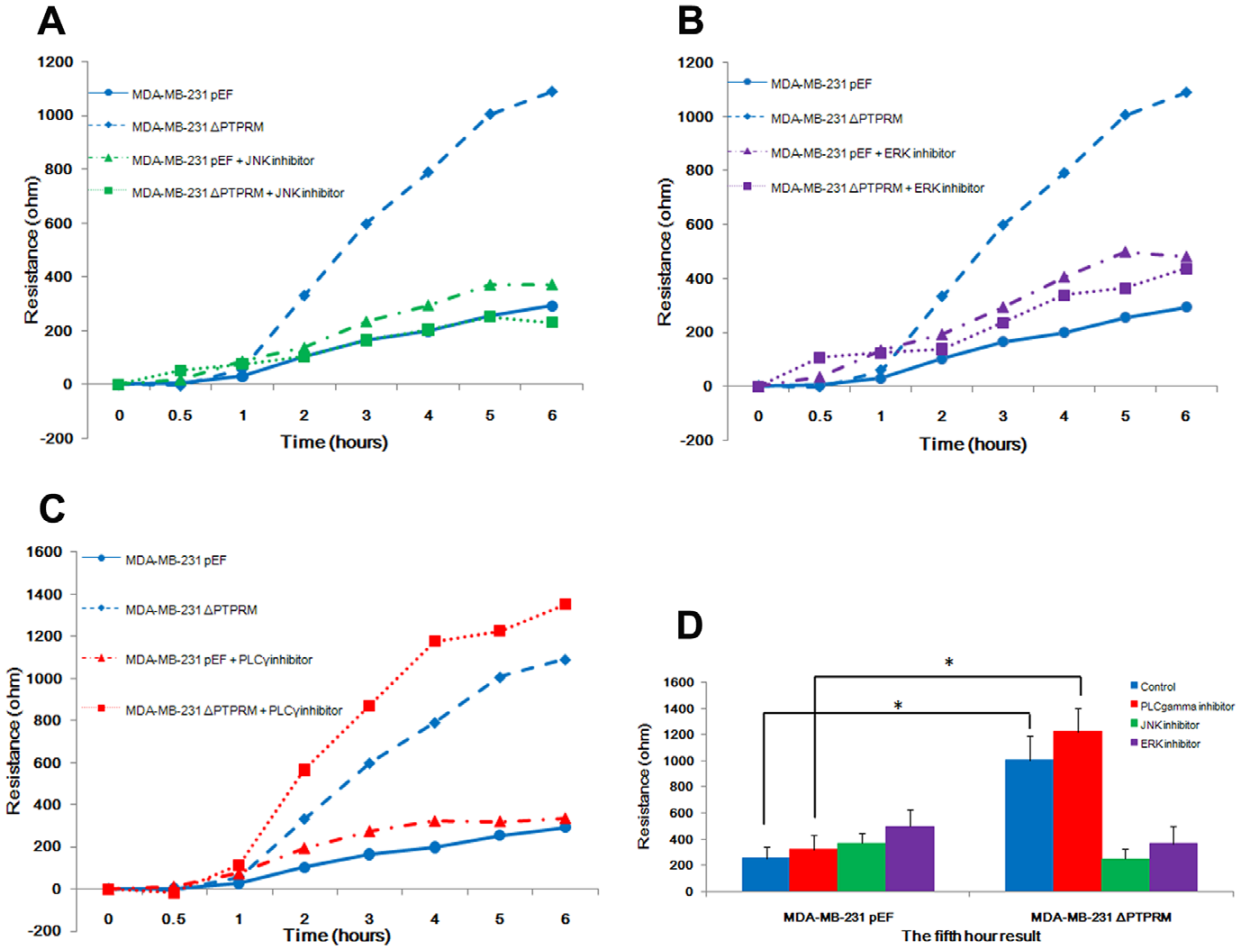
The knockdown of PTPRM in MDA-MB-231 cell resulted in increased cell motility via JNK and ERK pathways. A, *in vitro* wounding assay showed that MDA-MB-231^ΔPTPRM^ cells promoted cell migration. B, incubation of MDA-MB-231^ΔPTPRM^ cells with PLCγ small inhibitor had no effect on cell migration using ECIS. Incubation of MDA-MB-231^ΔPTPRM^ cells with JNK small inhibitor (C) and ERK small inhibitor (D) diminished such effect. E, the overall changes of resistance on the fifth hour with statistical analysis. *, *p*<0.05 and ***, *p*<0.001.

**Figure 7:**
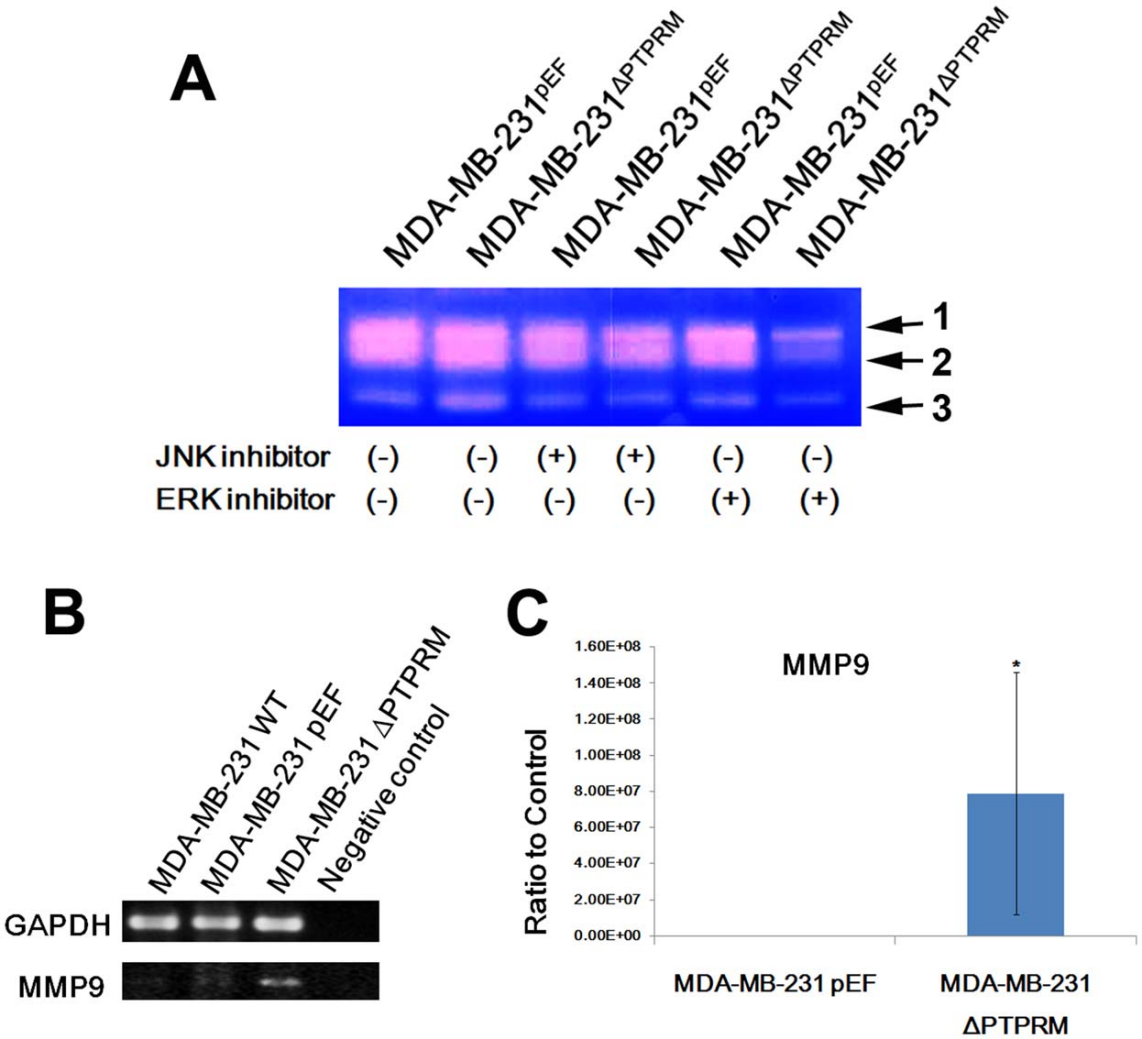
MMP9 expression and activity in MDA-MB-231 cell. The overall MMP9 gene expression was increased in MDA-MB-231^ΔPTPRM^ cells using (A) RT-PCR and (B) real-time quantitative PCR. C, gelatine zymography indicated the reduced emzyme activity of MMP9 in cells treated with ERK inhibitors. 1: pro-MMP9, 2: MMP9, and 3: MMP2. *, *p*<0.05.

**Figure 8:**
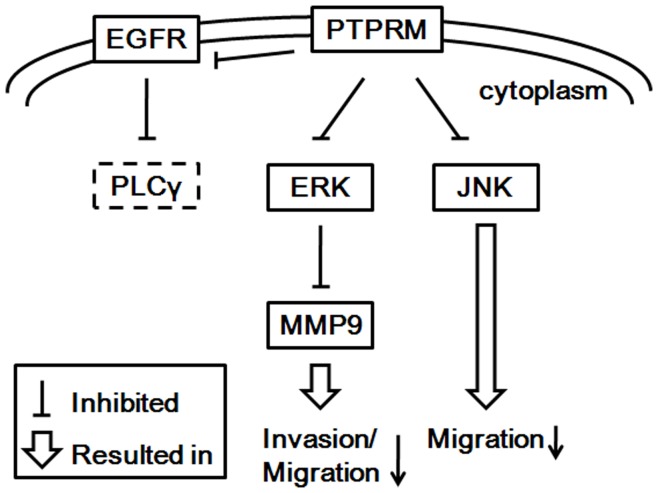
Potential interacting pathways and molecules involved in the functions of PTPRM in breast cancer cells.

### Real-time Quantitative PCR

The level of PTPRM transcripts in the breast cancer cohort was determined using a real-time quantitative PCR, based on technology which was modified from a method reported previously [21]. Primer sequences are shown in **Table 1**. The reaction was carried out on an IcyclerIQ™ (Bio-Rad, UK) which is equipped with an optical unit that allows real-time detection of 96 reactions. The reaction conditions were: 94°C for 12 min, 90 cycles of 94°C for 15 sec, 55°C for 40 sec (the data capture step) and 72°C for 20 sec. The levels of the transcripts were generated from an internal standard that was simultaneously amplified with the samples.

### Construction of Ribozyme Transgene Targeting Human PTPRM and the Establishment of Corresponding Stable Transfectants

Anti-human PTPRM hammerhead ribozymes were designed based on the secondary structure of the gene transcript and generated using the Zuker RNA mFold program [22]. The ribozymes were synthesized and then cloned into a pEF6/V5-His TOPO vector (Invitrogen, Paisley, UK). The verified ribozyme transgenes and empty plasmids were transfected into MDA-MB-231 (MDA-MB-231^ΔPTPRM^ and MDA-MB-231^pEF^) and MCF-7 (MCF-7^ΔPTPRM^ and MCF-7^pEF^) cells, respectively using an Easyjet Plus electroporator (EquiBio, Kent, UK). After a period of selection with 5 µg/ml blasticidin (up to 10 days), the verified transfectants were cultured in maintenance medium containing 0.5 µg/ml blasticidin. Primer sequences of the ribozymes were 5′-CTGCAGCCTGTCTCCTGCCACGGTCCTGCCCTGATGAGTCCGTGAGGA-3′ and 5′-ACTAGTGCTACCTTCCAGTGCAGTGCCATTTCGTCCTCACGGACT-3′.

### Cell Growth Assay

Breast cancer cells (3,000 cells/well) were plated into 96-well plates. Cells were fixed in 4% formaldehyde after 1 and 3 days of culture [23]. The cells were then stained with 0.5% crystal violet. Absorbance was then determined at a wavelength of 540 nm using a spectrophotometer (BioTek, ELx800). Growth rate of Day 3 (%) = absorbance of day 3/absorbance of day 1×100.

### Cell Matrix Adhesion

Cells (20,000) were added to each well of a 96-well plate which was pre-coated with Matrigel (5 µg/well) (BD Biosciences, UK). After 40 min of incubation, non-adherent cells were washed off using BSS buffer. The remaining cells were fixed with formalin and stained with crystal violet. The number of adherent cells was then counted under a microscope.

### In vitro Invasion Assay

This was performed as previously reported and modified in our laboratory [24]. Briefly, each transwell insert (upper chamber) containing 8 µm pores was coated with 50 µg of Matrigel and air-dried. The matrigel was rehydrated before use. 20,000 cells were seeded into each insert. After 72 hours incubation, cells that had invaded through the matrix and adhered on the other side of the insert were fixed and stained with crystal violet. The number of invaded cells was then counted using a microscope.

### In vitro Migration/wounding Assay

Cells were seeded into a 24-well plate and allowed to reach confluence. The cell monolayer was scraped using a fine gauge needle [25]. Photos were taken at 0.25, 1, 2, 3, and 4 hours after wounding. Migration distances were measured using ImageJ software (National Institutes of Health, USA).

### Electric Cell-substrate Impedance Sensing (ECIS)

The ECIS system (9600 model, Applied Biophysics Inc., USA) was used to quantify cell migration as previously reported [26]. 96W1E arrays were used in this study. MDA-MB-231^pEF^ and MDA-MB-231^ΔPTPRK^ cells were seeded at 40,000 cells per well in 200 µl of DMEM medium alone or medium supplemented with 200 nM PLCγ (U-73122), JNK (SP600125), ERK (FR180204) small inhibitors (MERCK, Germany). The resistance at 30 kHZ was recorded for 6 hours after wounded, and data was analysed using an ECIS-9600 software package.

### Immunoprecipitation (IP) and Western Bolt Analysis

Protein was extracted from 75 cm^2^ flask which were initially seeded with 4×10^6^ cells and cultured overnight. The protein samples were incubated with primary antibodies (**Table 2**) at 4°C for 1 hour then incubated for another hour after the addition of conjugated A/G protein agarose beads (Santa Cruz Biotechnology). The samples were washed twice with SDS-free lysis buffer before being boiled with 1x sample buffer (Sigma-Aldrich Inc, USA).

Equal amounts of protein were separated by SDS-PAGE and blotted onto nitrocellulose membranes. The membrane was then probed with the respective primary antibodies and corresponding peroxidise-conjugated secondary antibodies. Protein bands were visualised using a chemiluminescence detection kit (Luminata, Millipore) and photographed using UVITech imager (UVITech, Inc.).

### Gelatin Zymography Assay

1×10^6^ cells were counted and seeded to a tissue culture flask and incubated overnight. Following incubation, samples were washed once with 1xbalanced salt solution followed by a wash with serum-free DMEM and then either incubated in serum-free DMEM control or treated medium for 4 hours. The treatments consisted of a 200 nM JNK or ERK inhibitors (MERCK, Germany). After 4 hours the conditioned medium was collected. Protein samples were prepared in non-reducing sample buffer (0.625 mM Tris-HCl, 10% glycerol, 2% SDS, and 2% bromphenol blue). Samples were separated using SDS-PAGE on gels containing 1% gelatine (Sigma-Aldrich Inc, USA). Gels were renatured for 1 hour at room temperature in washing buffer (2.5% triton X-100 and 0.02% NaN_3_) and incubated at 37°C in incubation buffer (50 mM Tris-HCl, 5 mM CaCl_2_, and 0.02% NaN_3_) for 36 hours. The gel was stained with coomassie blue. The brightness of clear bands, where MMP9 was located and gelatine was degraded, was analysed using densitometry.

### Statistical Analysis

Statistical analysis was performed using SPSS18 (SPSS Inc., Chicago, USA). A Mann-Whitney U-test was used for analysis of non-normally distributed data, while the t-test was used for normally distributed data. Survival data was analysed using the Kaplan-Meier survival analysis. *p*<0.05 was considered statistically significant.

## Results

### Expression of PTPRM in Breast Cancer

A lower level of transcript expression was seen in breast cancer cells and the breast cancer tissue compared with normal mammary background tissue. Although transcript levels of PTPRM appeared to be reduced in breast cancer tissue in comparison with normal mammary tissues, the difference was not statistically significant (**Figure 1**).

### Association of PTPRM with Tumour Grade and TNM Staging

Levels of PTPRM transcripts were analysed against the corresponding clinical and pathological data (**Table S1**). PTPRM levels were higher in well differentiated tumours and were decreased in moderately differentiated tumours (*p* = 0.011) and poorly differentiated tumours (*p* = 0.031) (**Figure 2A**). Furthermore, PTPRM levels were higher in tumours of early TNM stage and decreased in the TNM2 (*p* = 0.032) (**Figure 2B**). TNM3 and TNM4 tumours also tended to have decreased levels of PTPRM, compared to TNM1 tumours (**Figure 2B**), but this was not statistically significant. This lack of significance was likely due to a smaller number of samples at advanced stages (TNM3, n = 7 and TNM4, n = 4). Tumours with lymphatic involvement appeared to have lower levels of PTPRM transcripts than the node negative tumours but was not statistically significant (**Figure 2C**).

### Reduced PTPRM is Associated with Poor Prognosis

According to the Nottingham Prognostic Index (NPI), we further analysed the relationship between prognosis and PTPRM expression (**Figure 2D**). Patients with moderate prognosis (NPI 3.5–5.4, *p* = 0.39) and poor prognosis (NPI>5.4, *p* = 0.06) exhibited lower levels of PTPRM compared to the good prognosis group (NPI<3.5). According to clinical outcome from our follow-up data, PTPRM transcript levels were decreased in patients who died from breast cancer when compared with that of disease free patients (*p* = 0.012) (**Figure 2F**). To investigate whether PTPRM expression levels were correlated with long-term survival, we divided the patients into two groups according to the average PTPRM transcript levels of patients with moderate prognosis of NPI 3.5–5.4. Kaplan-Meier survival analysis demonstrated that the expression of PTPRM transcripts was significantly associated with disease free survival (**Figure 2G**). The patients with lower expression of PTPRM had shorter survival (median = 109.5 months, 95% CI = 94.1–124.9), *p* = 0.029 vs. that of patients with higher expression levels (median = 142.5 months, 95% CI = 129.6–155.4 months).

### Knockdown of PTPRM in Breast Cancer Cells

The expression of PTPRM was knocked down using ribozyme transgenes targeting human PTPRM mRNA. Two different breast cancer cell lines were employed in the current study. MDA-MB-231 cells are oestrogen receptor (ER) negative and appear to be more aggressive compared to MCF-7 cells which are ER positive. The knockdown of PTPRM in these two cell lines was verified in the transfectants using RT-PCR, real-time quantitative PCR and western blot (**Figure 3**). Marked reduction of PTPRM expression was seen in both MDA-MB231^ΔPTPRM^ and MCF-7^ΔPTPRM^ cells which were transfected with ribozyme transgenes, compared to their corresponding wild type and empty plasmid controls.

### Effect on Breast Cancer Cell Functions by Knockdown of PTPRM

The effect on *in vitro* cell functions (cell growth, adhesion, invasion and migration) by PTPRM knockdown was examined. Knock-down of PTPRM in both MDA-MB-231 and MCF-7 cells exhibited a dramatic impact on *in vitro* cell growth. Both of MDA-MB-231^ΔPTPRM^ and MCF-7^ΔPTPRM^ cells showed an increased growth rate at day 3 (compared with empty plasmid control (**Figure 4A and 4B**). Knock-down of PTPRM in both cell lines had a significant influence on cell matrix adhesion. Both cell lines exhibited a significantly stronger ability to adhere to matrix compared to empty plasmid control (**Figure 4C and 4D**). The invasiveness of both MDA-MB-231^ΔPTPRM^ and MCF-7^ΔPTPRM^ cells were also enhanced after knock-down of PTPRM. *In vitro*, both MDA-MB-231^ΔPTPRM^ and MCF-7^ΔPTPRM^ cells shown an increased invasive capacity compared with respective controls (**Figure 4E and 4F**). Finally, MDA-MB-231 cells, knock-down of PTPRM also enhanced cell motility (*p*<0.001) compared with MDA-MB-231^pEF^ (**Figure 4G**).

### Tyrosine Phosphorylation of JNK and ERK were Increased by PTPRM Knockdown

Recent reports have shown that a reduction in expression of Protein Tyrosine Phosphatase-1B (PTP1B) and T-Cell Protein Tyrosine phosphatase (TC-PTP) reduced ERK phosphorylation in MCF-7 cells [27], and PTPRM suppressed glioma cell migration by dephosphorylation of PLCγ1 [20]. To determine the relationship between these proteins, immunoprecipitation (IP) and western blotting was used to investigate the impact of PTPRM on the tyrosine phosphorylation of PLCγ1, JNK, and ERK in MDA-MB-231 cell (**Figure 5A**). Results showed that tyrosine phosphorylation of both ERK (**Figure 5B**) and JNK (**Figure 5C**) were increased in MDA-MB-231^ΔPTPRM^ cells compared with MDA-MB-231^pEF^ cells (*p*<0.05). Our data also suggests that there is no change in tyrosine phosphorylation of PLCγ1 in PTPRM knockdown cells compared to control cells (**Figure 5D**).

### Involvement of JNK and ERK Pathway in PTPRM Impact on Cell Migration

In order to investigate breast cancer cell migration we used the ECIS system to analyse cell motility. MDA-MB-231 cells were treated with 200 nM PLCγ1, ERK, and JNK small inhibitors. As shown in Fig. 6, a decrease in cell motility was observed in MDA-MB-231^ΔPTPRM^ following incubated with JNK (**Figure 6A**) and ERK (**Figure 6B**) small inhibitors compared to control cells, as shown by a slower rise in resistance compared to untreated knockdown cells, indicative of reduced migration onto the electrode. No effect was seen following the addition of PLCγ1 inhibitor (**Figure 6C**). **Figure 6D** **shows** the statistical analysis of resistance on the fifth hour following wounding and indicates that MDA-MB-231^ΔPTPRM^ cells migrated faster than MDA-MB-231^pEF^ cells in both control and PLCγ1 inhibitor treated groups, *p*<0.05. However, this effect on the migration was diminished in MDA-MB-231^ΔPTPRM^ cells when exposed to JNK or ERK inhibitors.

### Enzyme Activity of Matrix Metalloproteinase 9 (MMP9) was Increased via ERK in PTPRM Knockdown Cells

Recently several reports have shown that MMP9 expression and activity were up-regulated by the ERK signalling pathway in different human cells [28], [29]. In order to investigate MMP9 activity in breast cancer cells, we used gelatine zymography to analyse enzyme activity. Knockdown of PTPRM resulted in an increase of both active MMP9 and MMP2 in MDA-MB-231 cells, which was consistent with increased invasiveness. We further treated MDA-MB-231 cells with 200 nM JNK and ERK small inhibitor, respectively. The elevated MMP9 and MMP2 activity in PTPRM knockdown cells was reduced to a similar level seen in the control cells, especially after treatment with ERK inhibitor (**Figure 7A**). Furthermore, MMP9 gene expression is also increased in PTPRM knockdown cells (**Figure 7B and 7C**).

## Discussion

Previous studies looking at the different PTPs have, in general, indicated that increased expression of PTPs is associated with breast cancer [30]. This finding is not universal as Zheng et al [31] showed that PTPRG was more highly expressed in normal tissue than in breast tumour tissue. PTPRM appears to have a similar tumour suppressing role and, for the first time, we demonstrate that in breast cancer there is a significant reduction in transcript levels. In the current study, levels of PTPRM transcript in a breast cancer cohort were analysed against the corresponding clinical and pathological data. The results showed that patients with higher tumour grade had relatively lower levels of PTPRM expression. Furthermore, the patients with lower expression of PTPRM had shorter disease-free survival compared with those with higher expression.

Our present study has indicated profound roles played by PTPRM in breast cancer cells. Knock-down of PTPRM in both MDA-MB-231 and MCF-7 cell lines led to increased *in vitro* cell proliferation, adhesion and invasion. Currently, there is a study indicating that PKC is involved in PTPRM-dependent signalling. PTPRM, RACK1, and PKCδ exist in a complex in cultured retinal cells and retinal tissue. PKCδ is required for neurite outgrowth of retinal ganglion cells on a PTPRM substrate [32]. Furthermore, PTPRM regulates the PKC pathway to restore E-cadherin-dependent adhesion via its interaction with RACK1 [33]. Activation of PLCγ1 is associated with increased invasion of cancer cells. A recent study has shown that PLCγ1 is a target of PTPRM and dephosphorylation of PLCγ1 is a major pathway by which PTPRM suppresses glioma cell migration [20]. However, in our current study, no change in the activated PLCγ1 was seen in the PTPRM knockdown cells, and the promoted migration was not affected by a PLCγ1 small inhibitor. It suggests that the PLCγ1 pathway is not involved in the effect on these breast cancer cells by knockdown of PTPRM.

In addition to the PLCγ1 pathway, ERK and JNK pathways have been indicated in the functions of certain PTPs. It has been shown that reduction in expression of Protein Tyrosine Phosphatase-1B (PTP1B) and T-Cell Protein Tyrosine phosphatase (TC-PTP) reduced ERK phosphorylation in MCF-7 cells to regulate cell migration [27]. Inhibition of JNK and ERK1/2 reduced PTP1B protein expression [34] and the absence of PTP1B in endoplasmic reticulum (ER) resulted in an activation of JNK leading to a suppression of apoptosis [35]. Moreover, a 45kDa variant of TC-PTP (TC45) exits in the nucleus upon EGFR activation and inhibits the EGFR-dependent activation of JNK and consequent activation of PI3K/Akt pathway, but elicits no impact on activation of ERK2 [36]. To examine the involvement of ERK and JNK pathways in the effect of PTPRM knockdown on breast cancer cell motility, we treated the cells with small inhibitors for these pathways. The effect of PTPRM knockdown on cell migration was diminished by blocking these pathways individually. This indicates that the dephosphorylation of ERK and JNK by PTPRM plays a critical role in coordinating functions of breast cancer cells.

It has been indicated that MMP9 activity is related to the ERK pathway, as tumour necrosis factor (TNF) stimulates proMMP9 production in human chorionic trophoblast cells through ERK1/2 pathway but not JNK and p38 pathway [28] and miR-520c and miR-373 up-regulated MMP9 expression by activation of Ras/Raf/MEK/ERK in human fibrosarcoma cells [29]. Our data has thus indicated that MMP9 activity in MDA-MB-231^ΔPTPRM^ was increased compared with pEF control and this increase was diminished by the treatments with ERK and JNK inhibitors. MMP9 activity in the JNK inhibitor-treated PTPTM knockdown cells was reduced to a level similar to the corresponding control cells. This indicates that activation of JNK pathway resulting from PTPRM knockdown has contributed to the increased MMP9 activity. Surprisingly, in the ERK inhibitor-treated PTPRM knockdown cells, the MMP9 activity was reduced to a level lower than the control cells and that this appeared to be much less affected by the small inhibitors. This may be due to the dual actions by the ERK pathway on the MMP9, i.e. activation and transcriptional regulation. It also suggests that the activity of MMP9 tends to be more dependent on the MAPK pathway in the PTPRM knockdown cells. Additionally, the potency and unknown effect of the small inhibitors used in the current study may also be a reason for such a difference. However, the exact mechanism and other unknown interacting molecules involved in this effect require further investigation.

### Conclusions

In summary, results from this study suggest lower expression levels of PTPRM to be a characteristic of breast cancer. Lower expression levels of PTPRM are correlated with poor prognosis and reduced disease free survival. Moreover, knockdown of PTPRM resulted in elevated adhesion, invasion, and proliferation of breast cancer cells. Activation of ERK and JNK by tyrosine phosphorylation and consequent elevated MMP9 activity is involved in increased cell migration and invasion by PTPRM knockdown (**Figure 8**).

## Supporting Information

Table S1 Transcript levels of PTPRM in breast cancer.
(DOC)
