# Correction: Protein Tyrosine Phosphatase µ (PTP µ or PTPRM), a Negative Regulator of Proliferation and Invasion of Breast Cancer Cells, Is Associated with Disease Prognosis

**DOI:** 10.1371/annotation/c8259769-10d5-4acf-be8e-ba24ff22a3e5

**Published:** 2013-06-13

**Authors:** Ping-Hui Sun, Lin Ye, Malcolm D. Mason, Wen G. Jiang

There were errors in the figure legends of Figures 2, 4, 5, 6, and 7. The correct figure legends are accessible at the following link: http://www.plosone.org/corrections/pone.0050183.001.cn.docx.

