# Supplementary material for: Protein Tyrosine Phosphatase µ (PTP µ or PTPRM), a Negative Regulator of Proliferation and Invasion of Breast Cancer Cells, Is Associated with Disease Prognosis
Source: PLoS One. 2012 Nov 20;7(11):e50183. doi: 10.1371/journal.pone.0050183 (PMC3502354; doi:10.1371/journal.pone.0050183)
Supplement: Table S1 — Transcript levels of PTPRM in breast cancer. (DOC) [file pone.0050183.s001.doc]

**Supporting information**

**Table S1.** Transcript levels of PTPRM in breast cancer

| **Clinical/pathological features** | **Sample no.** | **Mean± SD (copy no.)** | ***P*-value** |
| --- | --- | --- | --- |
| Tissue sample |  |  |  |
| Normal | 33 | 1687±1337 |  |
| Tumour | 127 | 428.8±148.8 | 0.36 |
| Grade |  |  |  |
| 1 | 20 | 472±114 |  |
| 2 | 39 | 288.8±110 | **0.011** |
| 3 | 52 | 92.5±279 | **0.031** |
| NPI |  |  |  |
| 1 (<3.5) | 59 | 605.4±277 |  |
| 2 (3.5-5.4) | 35 | 339±132 | 0.39 |
| 3 (>5.4) | 15 | 83.8±25.7 | 0.06 |
| TNM |  |  |  |
| 1 | 61 | 423±105 |  |
| 2 | 36 | 172.8±45 | **0.032** |
| 3 | 7 | 212.7±208 | 0.27 |
| 4 | 4 | 165.5±109 | 0.13 |
| Clinical outcome |  |  |  |
| Disease-free | 81 | 510±209 |  |
| Poor outcome |  | 219±66 | 0.19 |
| With metastasis | 5 | 209±163 | 0.27 |
| With local recurrence | 4 | 432±298 | 0.84 |
| Died of breast cancer | 16 | 170±55 | **0.012** |
| Lymph node status |  |  |  |
| Negative | 59 | 605.4±277 |  |
| Positive | 50 | 257.7±91.9 | 0.24 |
